# Hek293 as a recombinant protein factory: three different approaches for protein production

**DOI:** 10.1186/1753-6561-9-S9-P74

**Published:** 2015-12-14

**Authors:** Leticia Liste-Calleja, Martí Lecina, Roland Schucht, Dagmar Wirth, Hansjörg Hauser, Jordi J Cairó

**Affiliations:** 1 Chemical Engineering Department, Universitat Autònoma de Barcelona, Barcelona, Spain; 2 InSCREENeX GmbH, Braunschweig, Germany; 3 Model Systems for Infection and Immunity, Helmholtz Center for Infection Research, Braunschweig, Germany; 4 Dept. of Gene Regulation and Differentiation, Helmholtz Center for Infection Research, Braunschweig, Germany

## Background

Recombinant products have reached the market in diverse areas, including pharmaceutical, veterinary food, pesticides and detergents. Noteworthy, approximately 30 recombinant products in the pharmaceutical sector account for more than 90 per cent of all recombinant product sales. Although microbial systems can be considered an attractive option for expressing certain biopharmaceutical proteins, many biopharmaceutical molecules are too large and complex and need to be expressed in mammalian cells.

The most reported cell lines used for industrial processes are CHO, NS0, Sp2/O-Ag14 and HEK293. In particular, the latter has been gaining significant importance in the biopharmaceutical field during the last two decades. While initially employed for adenoviral vector production HEK293 became also one of the preferred cell lines for transient or stable protein expression. This is mainly due to its high transfection efficiency. Meanwhile genetic manipulation also includes new techniques for directing the integration of the foreign gene to highly expressed chromosomal sites e.g. by RMCE.

**Figure 1:**
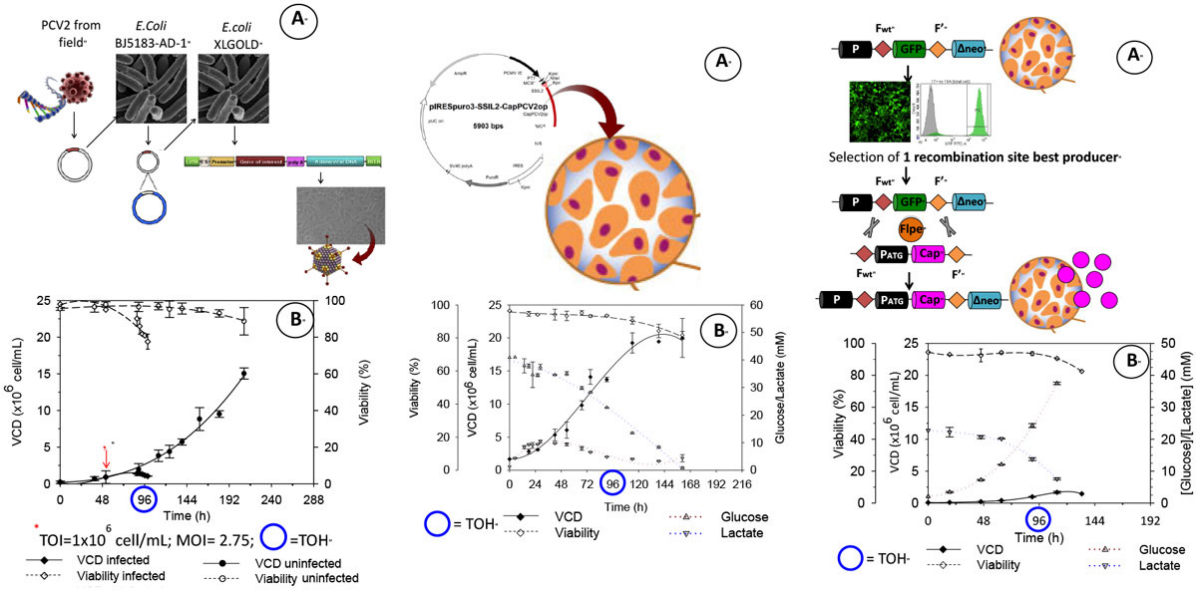
**Schematic representation of the three approaches studied for CapPCV2 protein production**.

In this work, three strategies for recombinant protein production in HEK293 cells have been compared (Figure 1): (St.1) rAdV production expressing protein of interest, (St.2) stable cell line establishment by random gene transfection or (St.3) site-specific integration using RMCE technology. As protein of interest we used the Cap protein from the capsid of porcine circovirus serotype 2 (CAP-PCV2). This virus is related to post weaning multisystemic wasting syndrome (PMWS), which has major implications for the pig industry worldwide.

## Materials and methods

### Strategy 1. CapPCV2 recombinant Adenovirus

Viral DNA was isolated from field and the gene of the capsid (CapPCV2) was cloned into Adh5 genome using AdEasyXL kit. Virus and protein production were performed by infecting HEK293-F6 suspension cells [1].

### Strategy 2. HEK293 stable cell line by random integration

CapPCV2 gene sequence was codon optimized for mammalian cell expression. The obtained sequence was cloned into pIRESpuro3 bicistronicvector [2]. HEK293-F6 suspension cells were transfected (PEI-DNA method) and positive cell pool was selected by puromycin addition to the media.

### Strategy 3. HEK293 stable cell line by RMCE

Three targeting vectors encoding for different promoters were transfected by electroporation to three different HEK293 master cell lines. Positive clones were selected by neomycin and ganciclovir addition to media. Clones were isolated by single colony pick up. Upon the selection of the best promoter, RMCE was applied to 293T cell line [3]. Positive clones were selected by puromycin addition to media and single colony isolation was carried out. The selected clone was finally adapted to grow in suspension.

## Results

We generated transient Cap-PCV2 producer cells by infection of the HEK293 cell line with a recombinant adenovirus (rAdV-Cap) (Figure 1, left panel). This approach gave a specific production (qp) of (41.291 ± 0.0002)e-3pg/cell, which is considerably low (Table 1).

**Table 1:** Values of significant process production parameters.

	Specific production (pg/cell)	[CapPCV2] (ng/mL)	Volumetric productivity (ng/(mL·day))
Adenoviral infection	(41.291 ± 0.0002)e^-3^	20.85 ± 0.84	9.25 ± 0.60
Illegitimate integration	0.107 ± 0.018	327.34 ± 62.53	103.14 ± 8.6
Site-directed integration	0.226 ± 0.034	256.57 ± 21.28	64.11 ± 0.13

In contrast, a significant increase in production was achieved upon random integration of a CapPCV2 encoding plasmid (Figure 1B) which resulted in a 2.5-fold increment of qp (0.107 ± 0.018pg/cell). The best results were obtained upon the site directed integration using RMCE technology (Figure 1C). Using this approach, CapPCV2 gene was inserted into previously identified chromosomal hotspots. The specific production of the RMCE-derived stable cell line (0.226 ± 0.034 pg/cell) doubled the one of the cell line obtained by illegitimate integration. Nevertheless, the highest volumetric productivity was calculated for the stable cell line after illegitimate integration (103.14 ± 8.6 ng/(mL·day)). This is related to the capacity of this cell line to grow in suspension in contrast to the adherent cell growth of the RMCE-derived cell line. The high cell densities achieved in cell suspension culture (≈18e6 cell/mL)[4]can compensate the lower qpof the cell line.

## Conclusions

The main conclusion of this work is that a bioprocess based on site-integrated stable suspension cell line would be the preferred one not only because the highest qp, but also due to the easy characterization of the process (in comparison to adenovirus infection), the repeatability of batch productions (in comparison to the other approaches) and the possibility of rapid substitution of the protein of interest.
